# *'It’s not going to be a one size fits all'*: a qualitative exploration of the potential utility of three drug checking service models in Scotland

**DOI:** 10.1186/s12954-023-00830-w

**Published:** 2023-07-27

**Authors:** Hannah Carver, Danilo Falzon, Wendy Masterton, Bruce Wallace, Elizabeth V. Aston, Fiona Measham, Carole Hunter, Harry Sumnall, Rosalind Gittins, Fiona Raeburn, Vicki Craik, Saket Priyadarshi, Laura Rothney, Kira Weir, Tessa Parkes

**Affiliations:** 1 Salvation Army Centre for Addiction Services and Research, Faculty of Social Sciences, University of Stirling, Stirling, FK9 4LA UK; 2 Canadian Institute for Substance Use Research, University of Victoria, Victoria, BC V8P 5C2 Canada; 3 School of Applied Sciences, Edinburgh Napier University, Edinburgh, EH11 4BN UK; 4 Department of Sociology, Social Policy and Criminology, University of Liverpool, Liverpool, L69 7ZR UK; 5 The Loop, Unclassified Community Interest Company, Manchester, M13 9PL UK; 6 Alcohol and Drug Services, NHS Greater Glasgow and Clyde, Glasgow, G51 1DP UK; 7 Public Health Institute, Liverpool John Moores University, Liverpool, L2 2QP UK; 8 Via, London, SW1H 9BL UK; 9 NHS Grampian, Royal Cornhill Hospital, Cornhill Road, Aberdeen, AB25 2ZH UK; 10 Public Health Scotland, Glasgow, G2 6QE UK; 11 Crew2000, Edinburgh, EH1 1PB UK

**Keywords:** Drug checking services, Harm reduction, Substance use, Drug use intervention, Drug related deaths, Qualitative research, Scotland, Public health, Service delivery

## Abstract

**Background:**

Scotland currently has the highest rates of drug-related deaths in Europe, so drug checking services are being explored due to their potential role in reducing these deaths and related harms. Drug checking services allow individuals to submit presumed psychoactive drug samples for analysis, and then receive individualised feedback and counselling. This paper explores participants’ views on the advantages and challenges of three hypothetical service models, to inform future service delivery in Scotland.

**Methods:**

Semi-structured interviews were conducted with 43 people: 27 professional stakeholders, 11 people with experience of drug use, and five family members across three cities. Vignettes were used to provide short descriptions of three hypothetical service models during the interviews. Interviews were audio-recorded, transcribed and analysed using thematic analysis.

**Results:**

Participants identified advantages and challenges for each of the three potential service models. The third sector (not-for-profit) model was favoured overall by participants, and the NHS substance use treatment service was the least popular. Participants also noted that multiple drug checking sites within one city, along with outreach models would be advantageous, to meet the diverse needs of different groups of people who use drugs.

**Conclusions:**

Drug checking services need to be tailored to local context and needs, with a range of service models being possible, in order to meet the needs of a heterogeneous group of people who use drugs. Addressing issues around stigma, accessibility, and concerns about the potential impact of accessing drug checking on access to and outcomes of drug treatment, are essential for successful service delivery.

**Supplementary Information:**

The online version contains supplementary material available at 10.1186/s12954-023-00830-w.

## Background

Scotland is experiencing a public health crisis in relation to drug-related deaths (DRDs) [1] There were 1300 DRDs reported in 2021, the highest rate in Europe at an age standardised rate of 25 deaths per 100,000 of the population [2]. The rate has increased by 500% from 1996 [2]. The increase in DRDs has been driven by a number of complex factors including high levels of deprivation, poly-drug use and an increasingly complex unregulated illicit drug market [2–5]. Benzodiazepines, largely non-prescribed novel benzodiazepines, have played an increasing role in this public health crisis [4] and were implicated in 69% of deaths in 2021 [2]. In Scotland, drug possession is a criminal offence under the Misuse of Drugs Act 1971 and drug policy is controlled by the UK rather than the Scottish Government.

The increase in DRDs has led to drug policy being placed higher on the national agenda, and there have been a number of subsequent policy and practical developments: the establishment of a Drug Deaths Taskforce in 2019 [6]; increased funding from the Scottish Government for drugs services and research [7]; publication of a National Drugs Mission Plan [8]; the expansion of naloxone administration programmes including carriage by Police Scotland, Scottish Ambulance Service and Scottish Fire and Rescue Service [9–11]; the establishment of a diamorphine (heroin)-assisted treatment service for a small cohort of people with opioid use disorder in Glasgow [12, 13]; the establishment of Medication Assisted Treatment Standards [14]; and ongoing policy work on the establishment of overdose prevention facilities and drug checking services (DCS), neither of which are currently operating in Scotland [1, 15–17]. Scotland has a strong harm reduction infrastructure, with widely available injecting equipment provision (IEP), take home naloxone, and opioid substitution therapy (OST) provision, which are a formalised part of Scottish Government’s strategy for reducing drug-related harms.

DCS are currently being explored in Scotland due to their potential utility and role in reducing DRDs [18]. These harm reduction services allow members of the public to anonymously submit (presumed) psychoactive drug samples for forensic analysis, with some services providing individualised feedback of results and counselling [19]. To be considered a drug checking service, drug samples must be collected directly from individuals and results and advice must be communicated directly to them, with the explicit aim of reducing harm [20, 21]. Whilst the first DCS were introduced in the USA in the 1960s, they have been in operation in European countries since the 1990s, originally in response to the increasing concerns around growing use of synthetic drugs as part of a burgeoning rave and night life culture [22–24]. The substantial increase in the number of DCS globally, has partly been driven by the growth in number and type of Novel Psychoactive Substances and the increased globalisation of unregulated drug markets [19, 25, 26]. Drug checking is increasingly recognised as an important tool for both reducing risk of harm to people who use drugs by providing information about the contents of a drug, and through drug market monitoring for public health purposes [26].

Despite many DCS sharing some key features outlined above, they differ widely in relation to how and where they operate [19]. A key distinction is between those which operate on a transitory basis at festivals and nightlife settings (known primarily as event-based drug checking) and those which operate on a permanent basis with individuals submitting samples directly for testing (known as community-based drug checking) [20, 24, 27–29]. Whilst DCS have often been seen as interventions aimed at those who use drugs within nightlife and leisure settings, there has been recent interest in community-based services which engage with those who carry the highest burden of drug-related harm [28, 30–33]. For example, in North America, DCS have been driven by the ongoing opioid overdose crisis [31], and are typically integrated into low-threshold settings such as overdose prevention sites and other harm reduction services [18, 34–37]. In Canada, a government funded pilot has seen the establishment of three DCS across Vancouver, Victoria, and Toronto [30, 38, 39]. Several services have also been established across the USA [18, 40, 41]. New Zealand is the first country to fully regulate drug checking with specific legislation for these services, with services operating across a range of fixed-site and mobile settings, including peer-led drug checking within IEP services [42, 43].

Whilst the increase of DCS globally has led to a subsequent diversification of service models and settings [19–21], there has been limited attention in the literature to different models of service provision and their suitability for different types/patterns of drug use. Studies have highlighted the need for DCS to be implemented in trusted, non-authoritative and low-threshold settings [28, 33, 44, 45], but there has been limited studies of different settings in which drug checking is offered, and how barriers and facilitators to engagement may differ across these settings. This paper addresses this gap by exploring the perceptions of Scottish stakeholders regarding the suitability of different sites and service models for the delivery of DCS to inform service delivery in three cities: Glasgow, Aberdeen and Dundee. These cities were chosen because they were keen to support the development of DCS and have some of the highest DRD rates in Scotland [2]. As there are currently no DCS in Scotland, participant views of three hypothetical settings were explored: a fixed site third sector (not-for-profit) setting, with a mobile van service; a service based in a National Health Service (NHS) drug and alcohol treatment service; and a service integrated in community pharmacy settings. These hypothetical settings were chosen by the research team following discussions about the potential locations for DCS based on how current harm reduction interventions are provided in Scotland. In Scotland, healthcare, including drug treatment, is provided for free at point of delivery. NHS services provide statutory drug treatment, and third sector services provide wider support services. Additionally, international examples of DCS were drawn on, to capture a range of current service models that would have potential in Scotland. While there are currently no pharmacy-based DCS, this was viewed as a potentially suitable option for Scotland because both IEP and opioid substitution therapy (OST) are provided in community pharmacies. These examples provided a starting point for discussion and participants were encouraged to make suggestions of other suitable locations for service delivery.

The aim of this paper is to explore participant perceptions of the advantages and challenges associated with each of the hypothetical service models. Further, a key question is the suitability of each model for different types/patterns of drug use, for example by people in nightlife and leisure settings who use more recreational drugs, versus those who experience marginalisation, who may be dependent, or exposed to higher risk drug markets.. Relatedly, this paper explores participant views on how to make drug checking accessible to a wide range of people who may wish to use the services. As well as contributing to the literature on this issue, the paper will inform implementation of DCS in Scotland by building knowledge on the suitability of different settings for service delivery. Other papers from this research have explored police participants’ views of drug checking [46] and key implementation barriers and facilitators [47].

## Methods

This paper reports the findings of a wider study conducted in Scotland to explore the potential for drug checking in the three cities. Ethical approval for the study was granted by University of Stirling’s NHS, Invasive and Clinical Research (NICR) panel (paper 0562; March 2021). NHS Research and Development approval was granted from each of the three NHS boards involved (for interviews with NHS staff only).

### Development of interview schedules and vignettes

As this paper was part of a wider project exploring DCS in Scotland, interview schedules (Additional file 1) were developed for each group and included a broad range of questions on issues such as the potential impacts of drug checking, stakeholders who should be involved in implementation and delivery, and contextual factors which may impact implementation or operation. This paper reports on participant views of three hypothetical service models, including levels of engagement with different groups of people who use drugs, and potential barriers and facilitators to engagement. As well as the interview schedules, vignettes were created which provided short descriptions of the three hypothetical service models (Additional file 2). They were developed by the research team based on existing drug checking models, information in the literature about preferred locations for drug checking amongst people who use drugs, and through consideration of potentially feasible locations for delivery in Scotland. These vignettes provided information about different models and enabled participants to discuss elements of service design and delivery in depth. The vignettes were provided to participants prior to the interviews, and in the interviews, they were asked about their views on each.

### Participant recruitment

Three groups of participants were involved: professional stakeholders (including those working in Police Scotland, NHS substance use services and third sector organisations); people who have used drugs in the last year; and family members of people who use/used drugs. These groups were chosen because of their expertise in the drug treatment, policy, and practice landscape and/or would likely be involved in, or affected by, the operation of DCS. Professional participants were eligible for the study if they were aged over 18 and worked in a relevant role in Aberdeen, Dundee, or Glasgow. Participants with experience of drug use were eligible if they were aged over 18, lived in one of the three cities and had used controlled drugs at least once in the last 12 months; while we attempted to identify those who use more recreational drugs, we were unable to interview anyone. Family member participants were eligible if they were aged over 18, lived in one of the three cities and were a family member of someone who had current/recent experience of drug use.

Participants were recruited through the research team’s networks and online searches for relevant organisations in each local area. Effort was made to ensure that all stakeholder groups comprised participants with a wide range of experiences and demographic features. For participants with experience of drug use, this included identifying more than 50 organisations working with a wide range of people who use drugs, as well as posting on harm reduction social media sites (including nightlife settings) and using the research team’s networks of those working in community organisations to recruit people not engaged with services. Professional participants included those working on the frontline in services as well as in managerial roles across a wide variety of services.

Written or oral informed consent was provided prior to each interview and participants were informed that they could withdraw from the study up to 48 h after the interview. Prior to the start of the interview, participants were asked questions about their age, gender, drug use, family relationship, roles, to obtain detailed demographic information. Interviews were conducted by telephone by DF/WM and ranged from 14 to 87 min in duration (mean 51 min). All interviews were audio recorded. Those with experience of drug use, and family members, were provided with a £20 voucher. Further details on the recruitment and interview procedure have been outlined elsewhere [47].

### Data analysis

Data were transcribed in full and analysed using NVivo12 (QSR International Pty Ltd., 2020). Transcripts were inductively coded using thematic analysis to identify themes [48]. One researcher (DF) conducted the initial coding, which was discussed and checked routinely by other members of the research team (WM, HC, TP). Sixteen transcripts (comprising a mix of the three participant groups) were coded to develop the initial coding framework, which was used as a template for analysis of the remaining transcripts. Coding of the remaining transcripts was conducted by WM and DF, who met frequently to discuss potential refinements or additions to the codebook. Finally, the data were sorted into themes and sub-themes, with all data relating to the service models written up separately for this paper.

## Findings

Forty-three interviews were conducted with professional stakeholders (*n* = 27), people with experience of drug use (*n* = 11), and family members (*n* = 5) across the three cities. Participant demographic details are provided in Table 1. Professional participants were aged between 27 and 64 years and worked in a range of roles, such as frontline worker, service manager and strategic decision-making positions. In terms of those with experience of drug use (PWEDU), they were aged between 34 and 59 years and most (*n* = 10) reported current or recent daily drug use. Poly-drug use was common and most used heroin (*n* = 8), non-prescribed benzodiazepines (*n* = 5) and powder and/or crack cocaine (*n* = 5). Most participants described infrequent engagement with drugs services. Affected family member participants were aged between 56 and 69 years and were parents of an adult son or daughter who used heroin or powder/crack cocaine on a daily basis.

**Table 1:** Participant demographics

Group	Total number	Gender	Ethnicity
Professional stakeholders	27	Female *n* = 14	White Scottish/British *n* = 26
		Male *n* = 13	White other n =1
NHS	9	Female n=8	
		Male n=1	
Third sector	8	Female n=4	
		Male n=4	
Police	10	Female n= 2	
		Male n=8	
People with experience of drug use	11	Female *n* = 3	White Scottish/British *n* = 11
		Male *n* = 8	
Family members	5	Female *n* = 4	White Scottish/British *n* = 5
		Male *n* = 1	
Total	43	Female *n* = 21	White Scottish/British *n* = 42
		Male *n* = 22	White other n=1

The findings are presented as two key themes. First, the perceptions of the advantages and challenges associated with each model are presented. Secondly, ways of expanding access to DCS are explored, drawing on the identified sub-themes of barriers to access amongst a diverse group of people, drug checking delivery across multiple sites, and outreach as a tool for increasing engagement.

## Exploring three potential settings for the delivery of drug checking

### Service model one—drug checking in a third sector setting

The first model presented comprised a service which delivered drug checking through a fixed site in a third sector setting which also had a mobile van which would facilitate access to drug checking across the city. While a specific third sector service was not proposed, these would be existing services who currently deliver support and harm reduction services to people who use drugs, including IEP. This was the most popular model with participants. The fixed site and mobile van are discussed separately.

#### Fixed site service model

The fixed site aspect of model one was generally popular amongst all participant groups. One perceived advantage of such a model was the pre-existing relationships and trust between many third sector organisations and groups of people who use drugs, particularly those who experience dependence and may be at high-risk of experiencing drug-related harm:

There is [third sector] hubs in every community… and most users would feel safer going near them than probably showing up at the [NHS] substance misuse service. (Participant with experience of drug use 4)

Trust was seen as a resource for encouraging people to engage with drug checking, given that drug possession is currently a criminal offence in Scotland. Related to the perceived trust between people who use drugs and third sector settings, such services were often described as less clinical and medicalised spaces, and as more rooted in the community, than NHS treatment services. One participant noted: *it’s less professional. It’s a bit more on ‘hi I’m your friend’ type of thing. I’m not here to judge you’* (Participant with experience of drug use 6). Another participant noted that a benefit of embedding drug checking in third sector services was that it was *away from the professionals and medication and all that sorts of stuff* (Participant with experience of drug use 5). A related benefit of the third sector discussed, particularly amongst participants with experience of drug use, was the potential for such settings to be staffed by peers: *more like one that’s got people with lived experience* (Participant with experience of drug use 8).

Although third sector settings were generally described as trusted, one participant highlighted that a third sector service in their city was viewed as too ‘close’ to statutory NHS treatment services, thus harming its credibility with some people and highlighting the need for careful consideration of services on a case-by-case basis:

They are too close to the treatment service. They are included in everything and people don’t trust them… So, it has to be something that people are going to trust. (Family member participant 2)

It was felt that people would be unlikely to use drug checking as a standalone service, but as part of a range of harm reduction supports. Several participants noted that the service hosting drug checking should also offer IEP, as this would provide opportunities for people who inject drugs to engage with drug checking:

It needs to be tied to an IEP site. I don’t think you should have them separately… because people still move, and they move to where they buy drugs and where they get their equipment. (Professional participant 11, NHS)

A further advantage of a third sector model is that it was perceived as a more relaxed and safer environment than NHS services, and whilst people waited for their results, they could access other supports and services:

Now if you want people to come to a fixed site to get their drugs tested and it takes half an hour or up to two hours, there needs to be something there for them… something as simple as… you provide a space where they come in, they go ‘oh hiya, there is my test, oh right it might take about half an hour today, cool I’ll just get a wee tea or coffee while I’m waiting then’. (Professional participant 4, third sector)

Although a third sector setting was generally seen as a good model for engaging a *subset of people who have a greater level of health and substance use need* (Professional participant 10, NHS), there were mixed views on its suitability for wider and varied groups of people who use drugs. It was highlighted that some prospective clients may not align themselves with a service which they perceived to be aimed at people who are dependent on drugs and use them on a daily basis, as is the case for many Scottish third sector drugs services:

Our injecting drug users, in an ideal world you would have a drug checking service where they come to get clean works, whereas, you know, I think you would be hard pushed to get a young person walking into a service like that who has maybe little or no knowledge of injecting drug use, but they want to use ecstasy at the weekend. (Professional participant 6, third sector)

However, not all participants agreed with this perspective. For example, one participant felt that if drug checking was framed as inclusive then there was no reason why people with different experiences and needs would not use the same service:

Just having it as open and as possible and, you know, just being as ‘this is for everybody, it’s not for one particular type of person’. And I suppose there is no reason why, you know, people wouldn’t mix. (Professional participant 9, NHS)

Similarly, some stressed the potential for third sector services to engage more ‘hidden’ groups of people who use drugs. Younger people engaging in more recreational use, or people who may be beginning to develop some challenges concerning their drug use, were seen as groups which a third sector-based model may be able to engage:

We have always struggled to get people involved at a younger age that are more starting out with so-called party drugs, ecstasy, and that kind of thing… and that would be great if this kind of service would attract them in earlier. (Professional participant 15, third sector)

Despite the general popularity of the fixed site model, participants discussed some challenges and limitations. Such settings were described as often having limited space, capacity, and resources, necessitating careful selection of a site based on adequate capacity. One participant described space and resource constraints in the third sector service which they attended, making such a setting, in their view, unsuitable for the delivery of drug checking:

The [third sector service] is too busy all the time… so there is not really much space in there, and there is a lot of things happening. I don’t think it would work in there. (Participant with experience of drug use 8)

Relatedly, some participants noted that the NHS could have an important role in providing resources, expertise, and training to third sector settings:

Yeah, I think the first one [the third sector model], is probably the one that you are going to get your best results from… maybe clandestinely having the NHS support them. (Professional participant 27, police)

This potential for a mixed model, drawing on the trust and legitimacy of the third sector and the resources and expertise of the NHS, was seen by some as a means of providing more extensive wrap around care and better links with treatment and other statutory services. Supporting this perspective, some participants described the benefit of having medical in-reach in third sector settings:

Maybe checking people… for abscesses and blood clots… because when I smoke crack cocaine I don’t know if I’ve got a blood clot on my lung …if you are in a drugs place like that, they know you are there to get checked… you are not going to be embarrassed. (Participant with experience of drug use 8)

#### Drug checking van service model

The second part of model one, a drug checking van operated by the third sector service who delivers the fixed site service, was generally popular amongst all groups of participants, despite the significant legal and security challenges that could be associated with such a model. The van option was particularly popular amongst participants with experience of drug use and family member participants, who noted its potential to increase access to DCS:

I think that’s a great thing, a marvellous thing… just the fact that they could come up to us, rather than us go to them. (Participant with experience of drug use 1)

Many participants felt that performing outreach drug checking could provide low threshold access for people with mobility or transport issues, those who were less inclined to engage with fixed site services, or those who lived outside of the city centre. Indeed, many family member participants and participants with experience of drug use described mobility issues as a barrier to engaging with services located in a city centre:

[A fixed site setting] will work well enough for people that are physically able to come to it. It might not work well for people who have mobility issues. My son does now, he can’t walk far. (Family member participant 1)

However, whilst the van option of model one was generally popular, participants discussed considerable challenges. Police participants noted potential concerns around people targeting these vans, as they felt that some people might try to break in and steal drugs, or to be involved in drug selling. In reality, only very small samples of drugs would be held on the premises and would be unfit for human consumption as a result of the testing process:

There is going to be people hanging around outside, you are also going to then attract people that are trying to source drugs… and see who is hanging around… so I can see complaints coming in about that from people that are seeing it happen. (Professional participant 21, police)

Another participant noted that, from their experience of a harm reduction van in their city, they can be quite an expensive and limited way of delivering a service:

I am not so keen… vans are quite an expensive way to do things and I think they have got limited use… you can’t get very many people in at a time, and they can’t be there all the time. (Professional participant 11, NHS)

Participants also reflected on the potentially negative perceptions of the wider community to such a service. It was noted that there may be a lack of support for mobile drug checking amongst local residents and the wider community:

I can see in terms of community response to a van pitching up in the street and a bunch of drug users coming along… I can imagine there would be an outcry from communities. (Professional participant 19, police)

### Service model two—drug checking in an NHS substance use service

The second model was the least popular model and was generally not seen as a viable stand-alone option. Participants stressed that some people who use drugs are often very mistrustful of NHS substance use treatment services due to previous negative and stigmatising experiences. One participant discussed their daughter feeling *‘*sick’ and ‘anxious’ when she had to attend the local NHS service:

My daughter hates going into that building, she hates it, because of the bad experiences she’s had. She is still, it still makes her feel sick. It still makes her anxious when she’s going in. (Family member participant 2)

NHS services were at times described as ‘cold’ and ‘clinical’ by participants. Relatedly, it was felt that people who are accessing NHS treatment for substance use may be unwilling to utilise drug checking in such a setting due to concerns over confidentiality and the effect of accessing such a service may have on their treatment, within a context of often abstinence-based NHS services:

I think people would be reluctant to engage with that because they are going to a service that they are using for provision of their drug use, care, and they would again be suspicious that that would be anonymous: ‘I am going to see you to get a methadone script and the conditions are that I am not using anything else’. (Family member participant 1)

Despite the significant challenges discussed in relation to service model two, participants did note some potential advantages. The presence of highly specialised staff within such settings was seen as an important resource. Participants also highlighted the pre-existing governance structures and protocols in statutory NHS services as a potentially useful resource for drug checking:

The NHS has a governance based, overarching, properly financed organisation which is the best organisation to coordinate and run these types of facilities. (Professional participant 8, police)

Whilst an NHS treatment service was not generally seen as a suitable location to host a standalone drug checking service, it was noted by some participants that it may be a useful addition to another model. Indeed, two participants with experience of drug use noted that they would likely access drug checking at their local NHS treatment service if it were available, explaining that the service was somewhere they were required to attend frequently, providing a convenient location for them to hand in drug samples for testing. One participant described the layout of their local NHS drug treatment service and noted that drug checking could be hosted in an extra room, run separately from the treatment service. They further highlighted that people would keep their treatment and drug checking separate: *you know people won’t come in with loads and they will be discreet* (Participant with experience of drug use 2). Further, some participants noted that treatment services providing drug checking could help the gradual process of rebuilding trust with people who use drugs in locations where there have been particularly fractious relationships:

It could work in the other way that people actually start to access it… therefore a drug checking service within the NHS… actually that rapport and trust becomes greater and changes the dynamic. (Professional participant 2, third sector)

### Service model three—pharmacy-based drug checking

The pharmacy service model was generally popular amongst participants who stressed its accessibility and the high levels of pre-existing footfall in such settings:

Heroin users will be going to the pharmacy anyway to pick up their methadone on a daily basis. (Family member participant 5)

Pharmacies offering IEP as well as OST were seen as the most suitable pharmacy sites for drug checking owing to the high levels of engagement amongst people who use drugs. A further perceived benefit of such a model was the large number of pharmacies in a city, aiding in the selection of strategic sites for implementation. The weekend opening hours of many pharmacies in these Scottish cities was also seen as a means of extending accessibility:

You’ve got a wider range of opening times. You’ve got your weekends as well which is sometimes when people do find it difficult because services are shut, so that is a plus point. (Professional participant 14, NHS)

Pharmacists’ level of specialism was seen as a potentially valuable resource for drug checking, with participants highlighting the potential for provision of tailored advice about drugs and their potential effects:

A lot of pharmacists, you can have a good talk with them, and they actually know what they are talking about… they actually know about what stuff is in it, they actually know what they are testing that for. (Participant with experience of drug use 9)

Despite the advantages of a pharmacy model, some potential challenges were discussed. Pharmacies were described as busy spaces, already offering a range of support and services and it was noted that drug checking would need to be adequately planned and resourced to ensure it was feasible given this. Related to resource constraints, some participants felt that integrating drug checking in a pharmacy could diminish the level of service provided to other customers, potentially causing tensions:

There is sometimes conflict between our people going in and then just other members of the public going in and the pharmacists having to sort of split their time or, you know, be in the small room doing supervised methadone or whatever. But then they are also needing to be elsewhere in the pharmacy to serve the other customers. (Professional participant 15, third sector)

Relatedly, participants also noted the importance of ensuring such a model was appropriately funded with adequate staffing: *It needs to be funded, it absolutely needs to be funded… has to be done by the pharmacist you are taking that pharmacist out of time for a while. So I think if there was a drug checking service available then it needs to be funded on time* (Professional participant 25, NHS).

Similar to service model two (NHS service model), participants noted concerns over confidentiality that may act as a barrier to engagement. Participants also questioned the level of discretion at some pharmacies. One family member participant described a local pharmacy setting, where people had to queue to receive OST and questioned the amount of discretion provided in such a setting:

I mean if you go down to [local pharmacy] in the town here, it’s in the main chemist area and there is a wee queue… for a wee hatch and that’s it, but they are queuing up into the main bit… and that doesn’t create confidentiality. (Family member participant 3)

However, other participants highlighted that pharmacies could be relatively discreet settings, as people could be attending to access any number of services or products and not just for drug checking:

I could be going to the pharmacy tomorrow to get a flu jab… I could be going to the pharmacy to get soap or sweets. (Professional participant 17, police)

Related to the differences in service provision across pharmacies, participants described mixed experiences of care from pharmacy staff. Participants described experiences of compassionate and psychologically informed practice as well as negative, stigmatising practice:

The pharmacy that I go to is really good, they are really helpful, and they always talk to you and make sure you are actually okay. Some pharmacies I’ve been in before, they know you are in for your methadone, so they want you out. (Participant with experience of drug use 7)

I know that some pharmacists are kind and compassionate, but there are a number who aren’t. That has been reported to me by my son… [he’s] been treated like dirt. (Family member participant 1)

The findings highlight that there are a range of potentially suitable service models for drug checking in Scotland, with third sector services and pharmacies likely being the most feasible. As well as debating the advantages and disadvantages of each service model, participants also described ways of expanding access to drug checking in Scotland, which will now be discussed.

## Expanding access to drug checking

Participants discussed a range of issues in relation to expanding the reach and accessibility of drug checking. Such discussions centred on the perception that people who use drugs are part of an extremely varied and heterogenous group, and that it would be challenging for drug checking in one site to be accessible and appropriate for all who may wish to use it. Participants also noted potential challenges relating to engaging in drug checking in different sites and fears around being seen by people they know:

I actually send my partner in to go and get [needles] because I don’t want people to see… Although it says it’s anonymous, people talk. And obviously everybody has got like a duty of care, especially with children. (Participant with experience of drug use 7)

Say someone that knew me saw me sitting there and didn’t know I took drugs. Most of my family know I take drugs but because I’m embarrassed that I take drugs… because for my family it’s embarrassing. (Participant with experience of drug use 6)

There was an overall view that drug checking is not a *one size fits all across this whole spectrum of people who take psychoactive substances* (Professional participant 10, NHS)*.* Therefore, drug checking in a single location was seen as unlikely to be equally accessible and appropriate for all groups of people. Participants felt that it would be beneficial to expand the accessibility and reach of drug checking as far as possible. Participants described two primary means of expanding access: implementation of drug checking at a combination of sites; and incorporating an outreach dimension to the service, which would involve samples being collected from a range of locations.

### Drug checking delivery across multiple sites

A significant proportion of participants (*n* = 18) discussed the need for drug checking to be implemented across a number of sites in order to provide options for people and overcome some of the barriers to engagement described above. The potential of implementing multiple drug checking service models was not something specifically asked or prompted in interviews; rather, such discussion was led by participants. Many participants expressed an ideal preference for some combination or hybrid of all three models: *there needs to be a mix of all three models* (Professional participant 13, NHS). A combination of a pharmacy-based model with a third sector run drug checking van was also frequently identified as a preferable approach: *the van and the pharmacy seem the best way* (Participant with experience of drug use 4). In addition to a combination of the models provided as examples, participants discussed alternative locations for the drug checking delivery. For example, one participant felt that drug checking could be located at a network of health centres throughout the city:

People have access to health centres within a very short walk, so some sort of service like this [drug checking] in the health centres. (Professional participant 16, police)

Others discussed the use of discreet drop-boxes in locations throughout a city as a potential means of increasing access:

I would put as many [drop-boxes] out as possible… put the drug you are interested in and if you want an answer phone this number with the serial number on the bag. (Participant with experience of drug use 2)

A number of participants with experience of drug use expressed a preference for safer consumption facilities/drug consumption rooms to be established in Scotland (there are currently no such services in operation), and for drug checking to be embedded within such settings:

In Germany they’ve got places where you can actually use your drug, that would be the safest place [drug checking] could be in Britain, but I don’t know why they won’t do it… they should have had it a long time ago. (Participant with experience of drug use 7)

Participants’ preference for a combination of models, and breadth of suggestions for potential locations, reflects a desire to see drug checking implemented as widely as possible to increase accessibility. For example, one participant with experience of drug use stated that they felt that drug checking should be *city wide* (Participant with experience of drug use 5). Echoing this, another participant stated:

So, it needs to be as cheap as it possibly can be and as much of it as it can be, so you are doing, you know, you can do three hundred tests in a week of all sorts of different [drugs] and different places, so you are getting really good live information out into the communities as quick as possible. (Professional participant 26, third sector)

### Outreach as a tool for increasing engagement

Outreach was described by many participants as a means of increasing engagement in drug checking. As explored above, in the discussion of the mobile drug checking van, outreach was seen as a means of low threshold provision for those who may not be able or willing to engage in fixed site services. Additionally, participants highlighted a range of other potential means of utilising outreach to expand access. One such means was having outreach staff collect and courier samples to DCS. It was noted that, despite likely legal and operational challenges, such an approach could significantly increase engagement. One participant noted that such an approach *could make sixty, seventy percent difference in the amount of samples you get to test* (Professional participant 4, third sector). Another form of outreach discussed was ‘in-reach’ testing, or sample collection in spaces such as emergency homeless accommodation and hostels:

If there was some way of doing drug checking outwith [beyond] your own service… like your emergency accommodations, your hotels with like sixty plus people in them. (Professional participant 5, third sector)

As one participant described, outreach approaches may provide significant benefits for those living outside of the city centre where harm reduction services were scarce:

[The wider city area] is huge and very rural so I think… [outreach] would increase your chances of people using it than if somebody had to get, say… two buses into [city]. (Professional participant 24, NHS)

Despite many participants discussing the importance of outreach drug checking or sample collection, it should be noted that there are significant logistical and legal issues associated with such an approach which make it unlikely to be implemented in the current context. However, participants did also describe more general uses of outreach which would be more feasible and pragmatic in the short-term. This included a role for outreach staff in speaking to groups of people who use drugs and helping to build trust and awareness:

The kind of things that are about just now like the [third sector organisation], they are putting the education out on the street because they are out there talking to the guys that are actually out on the street. (Participant with experience of drug use 11)

Overall, our findings highlight that there is the potential for a range of drug checking service models to be developed in Scotland, depending on local need and structures. Participants identified both advantages and disadvantages of the potential delivery of drug checking within third sector, NHS substance use treatment settings, and pharmacy settings, with NHS settings being the least preferred option. Participants also highlighted the need to consider a range of drug checking sites within one city, to meet the different needs of a heterogenous group of people who use drugs, with outreach models being identified as one way of increasing engagement.

## Discussion

This paper provides insight into the potential different service models available for drug checking in Scotland, exploring participant perceptions of the facilitators and barriers of different models, to inform future service delivery in Scotland. No DCS have been opened during the time the data were collected, although they are being developed in three cities. The findings of the research have informed the design of these services, with two cities choosing third sector services and the third a pharmacy, highlighting the needs for local needs assessments prior to implementation. Participants overall favoured the third sector fixed site/van model due to the perceived positive relationships that exist currently between many third sector organisations and people who use drugs, particularly those who are at high risk of drug-related harms, as well as identifying a range of potential barriers to implementation. The NHS substance use treatment service model was the least popular among all participants, due to a perception that many people who use drugs are mistrustful of NHS services as a result of negative past experiences in these settings. The pharmacy model was viewed positively by participants, who felt that it would likely be accessible to many people, due to convenient locations and opening hours, as well as pharmacists’ specialist knowledge. DCS would need to be delivered settings in the same way as other harm reduction services, such as IEP, for example with private spaces, different staffing, and separate data recording systems. There appears to be a need to increase trust in those settings where there might be concerns about confidentiality. There is also learning regarding providing DCS within drug treatment services (e.g., [52]) that can inform future service delivery.

Participants also reflected on other opportunities for delivering drug checking and how to ensure these services meet the needs of a heterogenous group of people. Delivering drug checking across a range of sites and using outreach were viewed as potential ways of increasing engagement. Participants noted that a combination of various aspects of each hypothetical service model would be advantageous, as well as having drug checking delivered in other settings, such as healthcare centres, discreet drop-boxes and within drug consumption rooms. Participants also felt that drug checking could be delivered using outreach workers, who would collect samples from a range of settings and deliver them to DCS for analysis. Having a range of options available was viewed as maximising engagement in DCS, meeting a range of people’s needs.

Our findings suggest that, for drug checking in Scotland, a ‘one size fits all’ approach is unlikely to be successful. Instead, settings need to be tailored to local context and needs, the different groups of people who might be accessing the services, their previous experiences and preconceptions, and current service provision, with local needs assessments and drug trends/toxicology data being used to help inform decision making on the most suitable model. These findings align with other studies regarding the accessibility of DCS: whilst community-based services receive multiple samples of drugs, they reach a relatively small proportion of people who use drugs [34, 49–51]. Participants in our study did not identify one approach as being the most appropriate but, instead, talked about a range of different options, including a combination of different approaches, service types and staffing. It should be noted that participants discussions tended to frame DCS as an intervention for those at higher risk of drug-related harm, due to the current levels of DRDs in Scotland. Whilst participants did, at times, discuss the suitability of the various models for wider groups, including those using recreationally, we were unable to capture these views directly. Therefore, it is important that future research explores the suitability of different service models with diverse groups of people who use drugs.

Studies of community-based DCS in England and Wales have highlighted a range of potential service models and the potential challenges associated with these. Measham [44] noted that some participants were reluctant to enter a drugs service for drug checking due to fears of being seen by family and friends, and due to the perceived stigma attached to the service. Instead, the service was provided via a church in a city centre location, which was viewed as most successful in engaging with a wide range of people who use drugs. In a pilot of a pharmacist-led drug checking service based in a third sector substance use service, it was noted that being based in a pre-existing service was beneficial, as was the ability to signpost individuals to other supports. Stigma was again reported as a potential barrier to accessing drug checking in a substance use service, although the authors reported that a range of people used the service, including those using drugs recreationally and family members [52]. Research from Canada has reported that a range of service settings is advantageous, including having central urban central locations alongside multiple distributed sites to provide access to drug checking in more rural areas [51]; in-person, remote and hybrid models [53]; and safe locations which do not put individuals at risk of criminalisation [30, 54]. Pharmacies, healthcare centres, and other community venues were identified as being settings which engage people with drug checking in a discreet manner, whilst also being relatively public and accessible spaces [30, 55]. Davis et al. [55] note that, in order to address stigma among people who use drugs, DCS should address people’s fears of being seen and have respectful staff. Embedding drug checking in wider community settings was viewed as a way of addressing the barrier of stigma [55].

Drug checking requires services that are non-stigmatising and welcoming to a wide range of people who use drugs, where they can feel comfortable to come and get their drugs tested without any judgement or repercussions from service providers and the wider community. Different service models are likely to be required due to the complexity of drug checking (i.e., equipment/testing process and legality) and the different needs of those accessing services, with outreach models and the availability of peers as ways of addressing barriers to engagement. Whilst it may be optimal for drug checking to be offered across a range of spaces to make them more accessible to the wide range of people who may wish to make use of such services, legal, logistical, and financial barriers may create challenges to realising this ‘distributed’ [51] or expanded model of drug checking. The equipment and staffing required for drug checking may also incur a significant cost to services and public health bodies, creating barriers to expanded models of drug checking. Research has highlighted a number of means of expanding access to drug checking and scaling up service provision at relatively low cost including remote sample spectra analysis (where drug checking equipment results can be interpreted remotely by technicians to reduce the need for expert staff on-site) [51]; having a number of points for sample drop-off throughout a city where samples can then be transported to a lab for testing [54, 56]; and the use of postal drug checking [28, 54]. Such approaches may face a range of legal and practical challenges. For example, sites may require licences, legal exemptions and building alterations to collect and transport samples, and postal drug checking may exist in a legal grey area depending on a country’s current legislative framework. Additionally, such approaches (particularly those based on provision of results via non-face-to-face methods such as online) may entail trade-offs such as fewer opportunities for the provision of wrap-around care and detailed in-person brief health interventions. Nonetheless, such approaches hold promise for expanding access to drug checking in a relatively cost-effective manner. In addition to providing more equitable access to drug checking technologies across rural and urban populations, expanding DCS through postal or other forms of outreach model could help build a wider and more detailed geographical picture of the drug market in Scotland, and how such trends may vary across regions. This could help inform the development and dissemination of more tailored and localised public health alerts and communications.

Organisations implementing and evaluating DCS should consider the needs and experiences of the target population when identifying appropriate service models and consider a range of options across cities, including the use of outreach. As this research was conducted at a time when no DCS existed in Scotland, more research is required to understand people’s experiences of different service models when services are established. Research is also required to examine how different service models are perceived within different legal frameworks. It is also important to address the stigma associated with drug use, drug services and drug checking to reduce barriers to engagement with services.

### Strengths and limitations

Data were collected from three groups of participants across three cities, providing a varied range of views and experiences of drug checking and service models. Whilst the data pertain to Scotland, findings are transferable to other countries with similar drug policies as Scotland and can inform future service delivery and research. As no DCS currently exist in Scotland, people’s limited knowledge and experience of drug checking may have limited their insight into these different options. Although many participants did have good insight into the types of service that could be feasible, we used vignettes to provide more detail on different service models to elicit richer views. Finally, we captured the views of 11 people with experience of drug use across three cities, but they were self-selecting and therefore their views will not reflect everyone who may use DCS. We were unable to identify people who use drugs in nightlife and leisure settings who may have had different views on the service models than our participants.

## Conclusions

This paper reports on the perspectives of people with experience of drug use, affected family members, and a range of professionals on the different potential service models of drug checking in Scotland. Different service models of drug checking are likely to be accessed by different people who use drugs, and it is important to consider key practical and logistical issues associated with these varied approaches. Fixed site settings were felt to offer a clear benefit of allowing trained staff to provide an enhanced harm reduction intervention to people who use drugs. Accessibility, stigma, and the perceived impact of accessing DCS on other aspects of care were identified as key barriers. A ‘one-size-fits-all’ approach to drug checking is unlikely to be successful and using different ways of engaging people in DCS is necessary.

## Supplementary Information

**Additional file 1.** Interview schedules for all groups

**Additional file 2.** Vignettes

## Data Availability

The datasets generated and/or analysed during the study are not publicly available. Individual privacy could be compromised if the dataset is shared due to the small sample involved.

## Abbreviations

DCS: Drug checking services
DRD: Drug-related deaths
IEP: Injecting equipment provision
NHS: National Health Service
OST: Opioid substitution therapy
