# Supplementary material for: 'It’s not going to be a one size fits all': a qualitative exploration of the potential utility of three drug checking service models in Scotland
Source: Harm Reduct J. 2023 Jul 27;20:94. doi: 10.1186/s12954-023-00830-w (PMC10373262; doi:10.1186/s12954-023-00830-w)
Supplement: Supplementary file 2 — Additional file 2. Vignettes [file 12954_2023_830_MOESM2_ESM.docx]

**Supplementary File 2. Vignettes**

**These vignettes have been developed to provide participants with information about drug checking. We will present three different service models and you will be asked your thoughts on each. We want to know what parts of the models you think would work in Scotland and which would not. They are not the exact models that would be used by services. The models are based on existing drug checking services but have been changed slightly.**

**Definition**

Drug checking services provide the opportunity for members of the public (and potentially services working with people who use drugs) to anonymously submit psychoactive drug samples for forensic analysis and subsequently receive individualised feedback of results alongside appropriate harm reduction information

**Background information**

There is some information which applies to all of the different examples of drug checking services discussed which it is important for you to know:

- All of the drug checking services would operate within a police tolerance zone, which means that people using the service wouldn’t be arrested whilst travelling to/from and using the service. This means that personal possession of drugs would not be criminalised in/around the services.
- Clients are not required to provide their name or address to the drug checking service. Staff at the drug checking service work under strict confidentiality. The services will ask clients to fill out a short, anonymous survey detailing only age, gender, ethnicity, location, occupation and patterns of substance use. If the client requires support in filling out this information, this can be provided by a member of staff.
- The services will test a range of drugs including benzodiazepines (like diazepam), opioids (like heroin), stimulants (like cocaine), psychedelics (like LSD). It will also test for many new psychoactive substances (previously known as legal highs) as well as common adulterants such as caffeine.
- Most services only require a small amount of the substance for drug checking (e.g. half a pill, third of a gram, residue from a bag), although some may need more for example a full pill.
- Samples which are handed in for analysis cannot be returned to the client.
- All facilities have a safe disposal option if a client wishes to dispose of their drugs.
- If a particularly dangerous or unexpected substance is found to be in circulation, through drug checking, this will trigger a ‘public health warning’ system. Information will be communicated through print media, social media, on harm reduction websites, through services that work with people who use drugs and through posters in key services and locations.
- Wider harm reduction advice is provided as part of drug checking services.
- Some services can provide very detailed information about the types of substances in the sample and the quantities, whereas other services provide quicker, less detailed information. The results can be provided more quickly for some substances than others (for example benzodiazepines will take longer than ecstasy).

**Model 1**

This drug checking service runs a mobile drug checking van and a service located in a third-sector-run harm reduction service. The fixed site drug checking is located in the city centre and the van travels to five different locations throughout the city, spending one day in each different area. Opening hours are Monday to Friday, 10am-5pm for both facilities. The mobile van has additional late-night hours on Thursday and Friday in the city centre, between 10pm-1.30am. The service is run by people with lived experience of drug use (peers), who have received specialised training in communicating drug checking results to clients. The staff are overseen by a specialist drug worker.

The sample required for checking is typically around a third of a gram, or half a pill/tablet for both sites. Drug checking at the van is done on the spot, and clients can receive their results within 30 minutes to two hours depending on how busy the service is (and the type of drug in the sample). Results of the drug checking are communicated in person.

At the service, clients also have the opportunity to deposit their sample in a secure drop box, but have to provide their phone number in order to be notified when results are ready. Drug checking is done off-site at a local laboratory, meaning that results will take between 1-2 days. Clients can either return in person to receive results, or can choose to receive a phone call from a member of staff who will communicate their results.

Both the van and the fixed site provide information about the contents of their sample and give support and advice around substance use and harm reduction including overdose awareness and how to minimise risks if they choose to take the drug following drug checking.

As this drug checking service is part of a harm reduction organisation, clients who use the van and the service are offered a number of other services and supports including: needle exchange and provision of safe injecting equipment, take-home naloxone, signposting to mental health and substance use organisations and housing support, and support with GP referrals. The organisation also runs harm reduction support groups to which those using the drug checking are invited to attend.

**Model 2**

This drug checking service is located within a NHS substance use service based in the city centre. Opening hours are Monday to Friday 9am-5pm. The drug checking service is run by a specialised team which includes chemists, pharmacists, substance use nurses and specialised harm reduction staff.

The sample required is small, a fingertip of powder or a small corner of a pill/tablet. However, the service encourages clients to submit more (up to a third of a gram or a whole pill) if they are able, as this helps to ensure accuracy of results.

Samples are deposited in a secure box and clients are given an ID number which they can use to get their results. This ID number can either be provided in person to receive results, or clients can download a confidential app which will notify them when their results are ready. The samples are analysed on-site by a specialised team, and the waiting time for results is typically 2-3 days. The service aims to reduce this waiting time to 5 hours within the next year.

Results are communicated by a pharmacist or substance use nurse and clients can only receive their results by returning in person. The information which a client receives is highly tailored to their patterns of drug use and can include information about overdose awareness and how to use substances safely.

As the drug checking service is part of a specialist substance use service, clients who identify concerns around substance use and/or physical and mental health can be referred into a range of services offered in-house, although use of these services is not a condition of using the drug checking service. The additional services include counselling, physical health check-ups and substance use support, including access to medication for drug use. The service also provides on-site access to take-home naloxone, needle exchange and safe injecting equipment.

**Model 3**

This drug checking service is located in a number of pharmacies throughout the city. Opening hours for all sites are Monday to Saturday 9am-5pm, and alternate Sundays 9am-5pm.

The sample size required is typically around a third of a gram or half a pill/tablet. As samples are transported off-site to a local lab for testing, waiting times are typically up to 3 days. Upon submitting a sample, clients are given a unique ID number which they can use to get their results. Clients can return with their ID number to receive results in person. However, this is not required and there are a number of other ways to receive results including through phone-call, through a confidential app, by email, text message or by logging into a website using their ID number.

Results in person are communicated by the pharmacist in a private space in the pharmacy. As well as information about the contents of their sample, this consultation can include tailored advice and support on overdose awareness and how to use substances safely. Clients who express concern about their use can be signposted to a range of substance use and other support services. If a client chooses to receive their results through written means (website, email, text message, app), then information is less tailored and includes only information about the contents of their sample. However, the written communication provides hyper-links to harm reduction websites and resources, including local services where people can get information and advice.
